# Quantification of threats to bats at localized spatial scales for conservation and management

**DOI:** 10.1371/journal.pone.0310812

**Published:** 2024-10-09

**Authors:** Brian M. Myers, Drew C. Stokes, Kristine L. Preston, Robert N. Fisher, Amy G. Vandergast

**Affiliations:** 1 U.S. Geological Survey, Western Ecological Research Center, San Diego, California, United States of America; 2 Department of Biology, Eastern Oregon University, One University Boulevard, La Grande, Oregon, United States of America; 3 San Diego Natural History Museum, San Diego, California, United States of America; University of Oklahama Norman Campus: The University of Oklahoma, UNITED STATES OF AMERICA

## Abstract

In a rapidly changing world, where species conservation needs vary by local habitat, concentrated conservation efforts at small spatial scales can be critical. Bats provide an array of value to the ecosystems they inhabit; many bat species are also of conservation concern. San Diego County, California, contains 22 of the 41 bat species that occur in the United States, 16 of which are on conservation watchlists. Thus, management of bat communities in San Diego County is a pressing need. Because bats exploit vast areas of the landscape and historical sampling strategies have shifted over time, a standardized way of prioritizing areas of the landscape for management would provide an integral asset to bat conservation. We leveraged long-term bat community survey data from sampling areas across San Diego County to prioritize areas with the most management need. We calculated two types of scores: species scores and threat scores. Species scores incorporated richness and conservation status, and threat scores included landscape level threats that bats could encounter. We found that urbanization, the presence of artificial lights, and areas sampled on unconserved land were all significantly associated with decreases in species richness. Further, using species and threat scores, each sampling area was placed into one of four conservation categories, in order from greatest to least conservation need, ranging from highest priority (high species score, high threat score) to lowest (low species score, low threat score). Additionally, we focused on sampling areas in which Townsend’s big-eared bat (*Corynorhinus townsendii*) and/or pallid bat (*Antrozous pallidus*) occurred. These two species are of exceptional conservation concern in San Diego County and across the western United States. We identified urbanization, the presence of artificial lights, and areas sampled on unconserved land as threats that were all significantly associated with the absence of Townsend’s big-eared bat, but not pallid bat. The strategy, methodology, and solutions proposed in our study should assist bat conservation and management efforts wherever bats occur, and can be extended to other species that require conservation attention.

## Introduction

Biodiversity conservation is a worldwide challenge: 33% of terrestrial vertebrate species are in decline, funding to address threatened species is limited, and the issues species face are multifaceted [1]. Some of the widespread threats species face across the globe include urbanization, climate change, and habitat fragmentation [2]. Further, the threats that a given species faces may vary based on local habitat type, increasing the complexity of conservation planning [3]. Fortunately, methods are available that can help identify localized areas of conservation need. For example, [4] provided a genomic approach to identify wild populations that require management attention. Additionally, [5] identified threats faced by loggerhead sea turtles (*Caretta caretta*) and designed generalized methodology that management can apply to any species with available demographic data. An array of effective conservation and management practices are required at localized spatial scales to accommodate diverse communities and to combat issues that vary by habitat. An important aspect of recently developed conservation practices is the potential for application to different species that occupy different components of the landscape, and the ability to apply conservation management at the local scale.

Conservation management in the United States is becoming more regionally coordinated, especially through the development and implementation of multispecies conservation plans [6]. These plans are essential tools in directing conservation and development over extended time horizons for communities, cities, counties, and states. For example, the San Diego Association of Governments’ Environmental Mitigation Program established the San Diego Management and Monitoring Program (SDMMP) to coordinate regional monitoring and management of species on conserved lands in western San Diego County [7]. The regional monitoring and management program encompasses multiple species conservation planning areas and supports and augments conservation efforts by many partners, including local jurisdictions enrolled in the plans, state and federal wildlife agencies, and non-profit conservation groups. The SDMMP developed the Management and Monitoring Strategic Plan for Conserved Lands in Western San Diego County, which identifies the need for a regional comprehensive bat management plan given the continued encroachment of human development on the bat community [7]. Ideally, management approaches will include strategies informed by biological data collection and monitoring of focal species and diversity trends. However, because management needs can differ by local habitat, it can be difficult to develop quantitative metrics to inform regional management strategies, especially from surveys conducted at much smaller spatial scales [8]. The difficulty of the transition from a general conservation plan to localized conservation action is compounded for species assemblages that are elusive and difficult to observe and monitor [9]. Approaches are needed to synthesize data from multiple sources and scales, both quantitative and qualitative. The current study focuses on bats (Order Chiroptera), a clade that includes many species in decline. We introduce a quantitative approach that can be applied to the local management and conservation of bat communities wherever they occur by leveraging species richness, ecological, and spatial data.

Worldwide, bats comprise about 25% of mammalian diversity, and play important roles in the ecosystems they inhabit [10]. For example, bats play a large role in insect population control, and about a third of bat species are important pollinators. [11–14]. Further, about 33% of bat species are fruit or nectar feeding and are thus pollinators and dispersers of numerous plants. In caves, bats are umbrella species that act as surrogates for cave biodiversity and conservation value and provide organic nutrients to cave ecosystems in the form of guano [15–17]. Despite the numerous ecosystem services bats provide, roughly 25% of bat species are threatened due to anthropogenic disturbance, lending urgency to bat community conservation and management [18, 19]. Bat communities exploit multiple parts of the landscape; thus, an effective management plan must incorporate landscape features used for roosting and foraging by bats within a given area. Roosting habitat is especially critical to bats [20], and some of these habitats, such as caves, mines, and bridges are especially prone to disturbance [17, 21]. Appropriate foraging habitat, such as open water and riparian land, is also needed to sustain bat populations and diversity [22]. Further, estimating species richness within a given sampling area can help prioritize management action to protect areas that house the greatest numbers of species [23]. In addition to the inclusion of richness data, species of bats with a conservation status may require prioritization [17]. The conservation issues facing bats are exacerbated due to the cryptic and vagile nature of these species and the numerous threats bats face as humans continue to encroach on their habitat [24].

California contains 25 of 41 bat species that occur in the United States [22, 25]. San Diego County, located within southern California, is a biodiversity hotspot for bats, and contains 22 species, 16 of which are included in conservation watchlists (Table 1 [26, 27]). San Diego County is comprised of predominantly semi-arid and arid habitats, and bats in these environments are poorly studied, increasing the difficulty of the identification of conservation needs [28]. San Diego County, from the western coastline to the eastern peaks of the Peninsular Range, contains over 273,000 ha of conserved lands [26, 27]. Lands under public ownership (e.g., United States Forest Service, California State Department of Parks and Recreation, and United States Bureau of Land Management) and adjacent undeveloped privately owned lands, tribal lands, and military lands support the diverse bat community in San Diego County. The diverse network of entities that own land in San Diego County can make management of bats a challenge. A single population of bats will utilize different parts of the landscape, so that protection of only one area used by bats could be inadequate. For example, bat species tend to have multiple roosting and foraging habitats in different areas. Thus, incorporating approaches to identify multiple areas of the landscape commonly used by bats, while also considering the severity of threats bats face across those areas could improve management and protection of the entire bat community [23].

**Table 1:** Bat species of California and sensitivity status.

Species	WBWG	CDFW
*Pallid bat (*Antrozous pallidus*)	H	SSC
*Townsend’s big-eared bat (*Corynorhinus townsendii*)	H	SSC
*California leaf-nosed bat (*Macrotus californicus*)	H	SSC
*Mexican long-tongued bat (*Choeronycteris mexicana*)	H	SSC
*Lesser long-nosed bat (*Leptonycteris curasoae yerbabuenae*)	H	SSC
Little brown bat (*Myotis lucifugus*)	M	
Arizona myotis (*Myotis occultus*)	M	SSC
*Yuma myotis (*Myotis yumanensis*)	LM	
Cave myotis (*Myotis velifer*)	M	SSC
*Long-eared myotis (*Myotis evotis*)	M	
*Fringed myotis (*Myotis thysanodes*)	H	
*Long-legged myotis (*Myotis volans*)	H	
*California myotis (*Myotis californicus*)		
*Western small-footed myotis (*Myotis ciliolabrum*)	M	
*Silver-haired bat (*Lasionycteris noctivagans*)	M	
*Canyon bat (*Parastrellus hesperus*)		
*Big brown bat (*Eptesicus fuscus*)		
*Western red bat (*Lasiurus blossevilii*)	H	SSC
*Western yellow bat (*Lasiurus xanthinus*)	H	
*Hoary bat (*Lasiurus cinereus*)	M	
*Spotted bat (*Euderma maculatum*)		
*Mexican free-tailed bat (*Tadarida brasiliensis*)		
*Pocketed free-tailed bat (*Nyctinomops femorosaccus*)	M	SSC
*Big free-tailed bat (*Nyctinomops macrotis*)	MH	SSC
*Western mastiff bat (*Eumops perotis californicus*)	H	SSC

Species found within San Diego County are indicated with an asterisk. Acronyms defined as follows: CDFW = California Department of Fish and Wildlife (SSC = Species of Special Concern) and WBWG = Western Bat Working Group (LM = Low-Medium Priority, M = Medium Priority, MH = Medium-High Priority, H = High Priority).

Bats face numerous threats in San Diego County, most of which affect the quality and availability of roosting and foraging habitat [22]. Within the rapidly developing region of San Diego County, roosting habitat is increasingly threatened by different forms of anthropogenic disturbance, including urbanization and habitat modification, human visitation, habitat fragmentation, and exclusion and extermination from private (and sometimes public) lands [29]. Threats to foraging habitat include lack of availability of open water and anthropogenic disturbance in the form of urbanization and habitat modification, human visitation, habitat fragmentation, pesticide use, artificial lights, and invasive species [9, 22, 30]. As urban and suburban development continues to expand throughout the foothills, interactions between humans and wild bat populations are compounding [31].

The goal of this study is to provide a regional data framework to support prioritized conservation planning and management strategies for bats within and nearby sampling areas throughout San Diego County. The current study compiles long-term data, includes measures of richness and conservation status, and identifies and quantifies potential landscape-level threats to bats. The use of long-term data can result in sporadic and uneven sampling thus we correct for uneven sampling in our dataset. We propose a prioritization framework based on bat diversity and threats to bats to guide conservation planning efforts for the protection of bat biodiversity throughout San Diego County.

The framework presented here provides a model for examining threats to bat communities wherever they occur, but also has the flexibility to adopt a species-specific approach. Townsend’s big-eared bat (*Corynorhinus townsendii*) and pallid bat (*Antrozous pallidus*) are two vulnerable species in San Diego County that are of high conservation concern [32, 33]. Townsend’s big-eared bat has experienced a reduction of available roosts, a reduction in the size of remaining colonies in California, and has experienced population declines over the last 40 years [34]. Roosts in mines, caves, culverts, flumes, and under bridges can be disturbed by human visitation and work projects by various agencies, such as transportation and water departments [32, 34]. The pallid bat has similar vulnerabilities as Townsend’s big-eared bat, and has experienced a range contraction, including the loss of many historical roost sites, and, like Townsend’s big-eared bat, might be undergoing a population decline [22, 32, 33, 35]. Because both Townsend’s big-eared and pallid bats are of conservation and management concern in San Diego County (and across their respective ranges), we further analyze our bat community management plan in the context of these two species.

## Methods

### Study area

**Fig 1:**
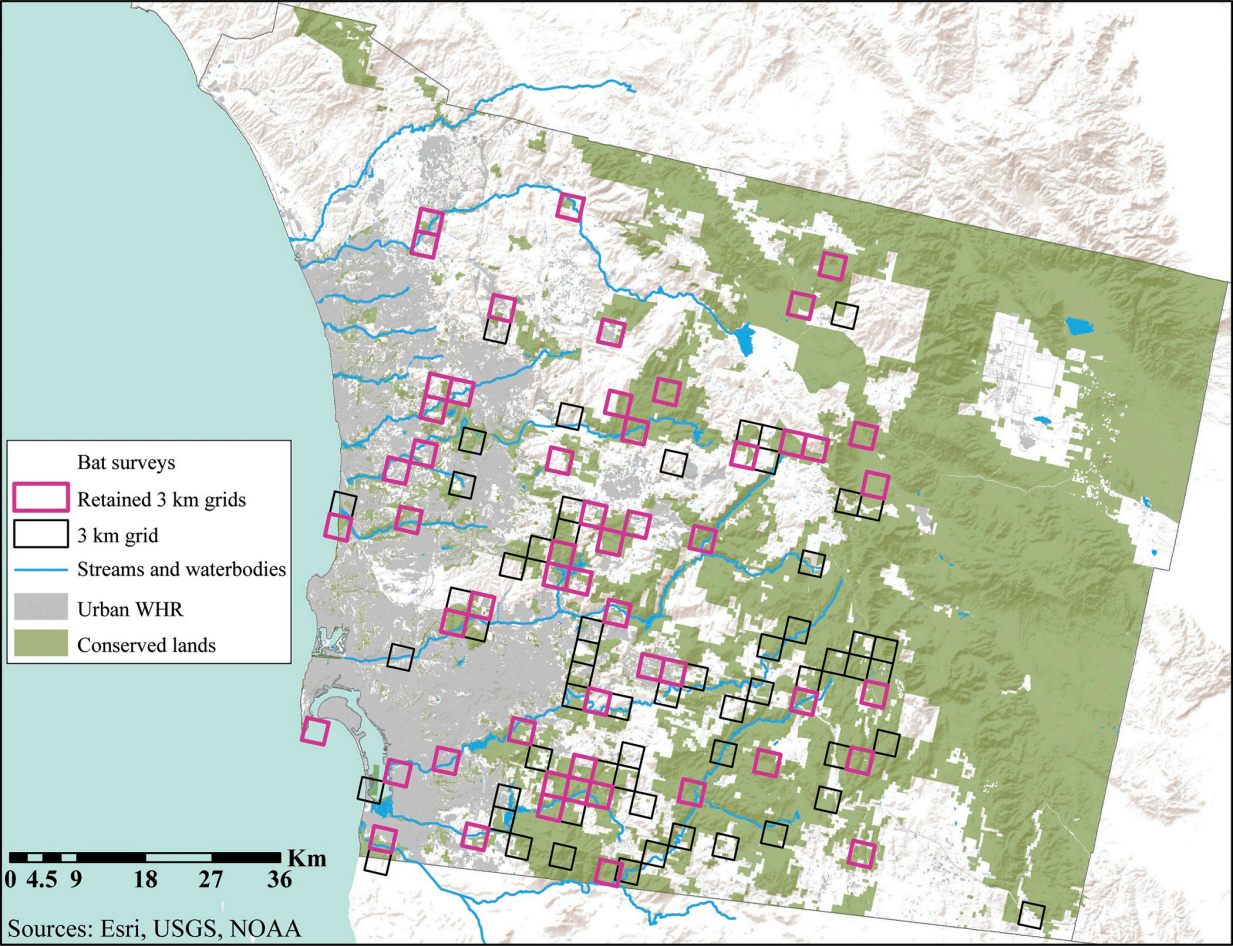
Locations of sampling areas, streams, and bodies of water in San Diego County, including all sampling areas considered for the study, and sampling areas that were analyzed after removing under-sampled areas [58]. Urbanized areas and conserved lands are also shown [55–57, 59].

We compiled bat survey data from 152 sampling sites throughout San Diego County in southern California from 2002–2019 (Fig 1, S1 Table, Stokes [Unpublished], [22, 36–42]). Survey methods included ANABAT bat detectors, the unaided ear, day roost surveys and exit counts, and night roost surveys. All surveys were led by or included D. Stokes. Surveys were conducted with permission from private landowners and public land managers in areas where species were historically known or had the potential to occur based on habitat preferences [39, 43, 44]. The study area in which surveys were conducted, San Diego County, spans a wide array of habitat types, including woodland, forest, coastal sage scrub, chaparral, and desert habitats, and apart from the desert, is generally characterized by a moderate Mediterranean climate [45, 46]. Elevation across San Diego County ranges from sea level along the coast to nearly 2,000 m in the mountains [46].

### Sampling

When bats were captured, captures were done using fine mesh mist nets (Avinet, Portland, Maine) and handheld butterfly nets. All surveys were conducted under the California Department of Fish and Wildlife permit SC#2645 and were approved by the Western Ecological Research Center Animal Care and Use Committee in association with the University of California, Davis. All statistics from survey data were calculated in R v3.5.2 [47] and R Studio v1.2.5 [48].

#### ANABAT bat detectors

1 to 7 detectors were deployed at 129 of the 152 sampling sites to record bat vocalizations in this study. Detectors were placed approximately one meter above the ground on T-posts or strapped to trees or vegetation with microphones oriented towards expected bat flyways. Expected bat flyways included the edge of riparian reaches, the edge of oak and/or coniferous woodlands, meadow edges, along scrubby ridgelines, and the edges of waterways. The detectors were left in place to passively record bat vocalizations for a minimum of one night. In rare cases, the detectors were left in place for an extended period (i.e, up to 31 consecutive nights at a sampling site in the Coronado Cays sampling area). The detectors were set to automatically turn on at sunset and turn off at sunrise. Detectors automatically recorded bat vocalizations as well as other sounds such as insect noise. After the detectors were retrieved from the field, the recorded bat vocalization data were downloaded and reviewed in the laboratory and identified to species level when possible [49]. All files were manually reviewed using multiple versions of AnalookW software (Titley Electronics, Queensland, Australia). No filters were used and all files were manually vetted during the review process.

#### Day roost surveys and exit counts

Diurnal inspections of known or potential bat day roosts were made opportunistically. Inspections involved peering into crevices and cavities where bats were visible, usually with a flashlight. Occasionally, bat roosts such as mines and flume tunnels were entered and bats were sometimes captured using a handheld butterfly style net to verify the species, age, sex, and reproductive status. On 112 occasions, bats were observed as they exited from a day roost. Bats were counted as they exited roosts using the unaided eye and clicker counters.

#### Night roost surveys

Inspections of sampling sites where bats were roosting at night were made opportunistically. A flashlight was used to illuminate night roosting bats so species status could be verified.

#### Fine mesh mist nets

Mist nets were erected in expected bat flyways including over small ponds, across creeks, in vegetation flyways, and under woodland canopies to capture flying bats [50]. All mist nets used were 2.6 meter-tall, single high-nets. Mist nets were used on 90 surveys. The number of mist nets used was dependent on the number of appropriate bat flyways at any given sampling site during a survey night. Mist nets were opened at approximately sunset and were continuously attended for three hours. After each three-hour mist netting period the nets were closed and taken down. Species, age, sex, and reproductive condition were determined for all captured bats. Most bats were photographed and all were released immediately after being processed.

#### The unaided ear

Listening for audible bat vocalizations with the unaided ears always accompanied mist netting but was also conducted independently of other survey techniques on 107 occasions.

### Spatial data and analysis

Because sampling locations were not evenly spaced, we overlaid a sampling grid of 3 km to group surveys that were geographically proximate and to extract spatial variables. Although home range varies, within California, average home ranges of several of the bat species within this study are estimated to be from 1–3 km, including Townsend’s big-eared bat and pallid bat [29, 51–54]. Thus, 3 km^2^ grid cells may approximate the area used by bats captured within them (*sensu* [24]). Bat species data were scored for a given site for species presence, conservation status, and landscape-level threats.

Landscape threats were calculated using the following GIS data layers: 1) Conserved Land database of parcels conserved for the purpose of protecting open space and natural habitats in San Diego County [55], 2) CalFire-FRAP FVEG vegetation layer [56], 3) Light pollution, Bortle 2 classifications [57], and 4) The National Hydrography Dataset Waterbody Feature Class [58, 59]. For conserved lands, urban wildlife habitat relationship type, and waterbodies, we calculated the proportion of each sampling area covered by the feature. For light pollution, we calculated the area-weighted average Bortle 2 score. For sampling sites that overlapped with the ocean, we excluded the ocean, so that total area was restricted to total land area.

### Threat score computation

We used GIS data to quantify landscape threats within a given sampling area. To calculate an unbiased threat score for each sampling area, raw values obtained from GIS data were standardized. For each threat, standardized values for each sampling area were scored based on the quartile in which they were placed for a particular threat. Scores ranged from zero to four points (one point for sampling areas scoring in the first quartile, two points for the second quartile, three points for the third quartile, and four points for the fourth quartile; Table 2). Each threat is described below. The biological impact(s) associated with each descriptor are identified in Table 2.

**Table 2:** Threat and species score computation.

Species	Points		Justification	
Richness	1 (each species)		1 point granted for every species detected	
Status	1 (each species)		1 point granted for every species with a conservation status	
**Threat**		**Impact on species**		**Scoring**
Artificial lights		Modification of prey availability		1 point if in quartile 1, 2 if in quartile 2, 3 if in quartile 3, 4 if in quartile 4
Pesticide use		Reduction of prey availability		
Unconserved land		Bats on unconserved land may be vulnerable		
Urbanization		Reduction of available/suitable habitat		
Lack of open water		Water is a critical resource to bats		

### Threat descriptions

For each sampling area, threats were quantified using GIS data as described below.

#### Artificial lights

The average light pollution score within a given sampling area, based on Bortle Class [57]. Due to their nocturnal nature, bats have a high probability of being affected by light pollution (S1 Fig, [60]).

#### Pesticide use

Based on the pesticide use map from the California Environmental Protection Agency for pesticide records from 2012–2014 [61]. The total number of pounds of a subset of 70 chemicals that were filtered for hazard and volatility were calculated to measure pesticide production. Production for each census tract was divided by each census tract’s area. Bats frequently forage in farmland and have high potential for pesticide exposure [62].

#### Urbanization

Calculated as the proportion of urbanized land area (not including the ocean) in a given sampling area, using the CalFire-FRAP FVEG layer. Bats are highly sensitive to urbanization [63].

#### Unconserved lands

The percentage of land that a sampling area occupies that is not conserved by a government agency or non-profit organization, including the 3 km buffer. The presence of legally protected land promotes the conservation of bats [19, 64].

#### Lack of open fresh water

Calculated by combining data from stream and open water layers as a proportion of land area within a sampling area. Open water sources are critical for bats; they rely on these sources for drinking and foraging [65].

### Species richness estimates and sampling effort

To investigate potential sampling bias, we added the number of surveys within each sampling area as a variable in a Generalized Linear Model (GLM). We incorporated a GLM to investigate which variables, including sampling effort, were significantly associated with species presence across sampling areas. Other variables we included in the GLM were potential threats that the bat community faces in San Diego County (described above), enabling investigation of which threats were significantly associated with the presence or absence of bat species. However, number of surveys was not included in calculation of the threat score for any sampling area. Further, uneven sampling effort can bias results, i.e. more species are likely to be discovered with increased sampling effort. Thus, to estimate whether each sampling area was adequately sampled, we constructed species accumulation curves of the cumulative total number of species that were identified as a function of the number of times a sampling area was surveyed, with the ’specaccum’ function within the "vegan" R package [47, 48]. Species accumulation curves were made for each sampling area with more than one survey. The chao, jack1, jack2, and boot metrics estimated how many species were actually present at each given sampling area based on the number of species observed and the number of surveys conducted (S2 Table). Each of these metrics are widely used nonparametric richness estimators and have varying degrees of reliability (i.e., they produce different levels of type-I error rates) and no single metric clearly stands out as the most effective [66]. Thus, we evaluated each of these metrics. Because infinite sampling effort is impossible to achieve, we analyzed data from sampling areas in which at least 80% of species were sampled, while sampling areas below this threshold were omitted from the final dataset [67].

### Species richness and species score computation

Conservation status, in addition to species richness, can assist in guiding management [17]. By using a species richness score weighted by conservation status, both the overall number of species and species of conservation importance are reflected in the score [17]. We scored species at a given sampling site as follows: one point for every species detected, and an additional point for each species listed as a California Department Fish and Wildlife Species of Special Concern (SSC, Table 2). Species scores were calculated based on the observed number of species within a given sampling area.

### Conservation categories

**Fig 2:**
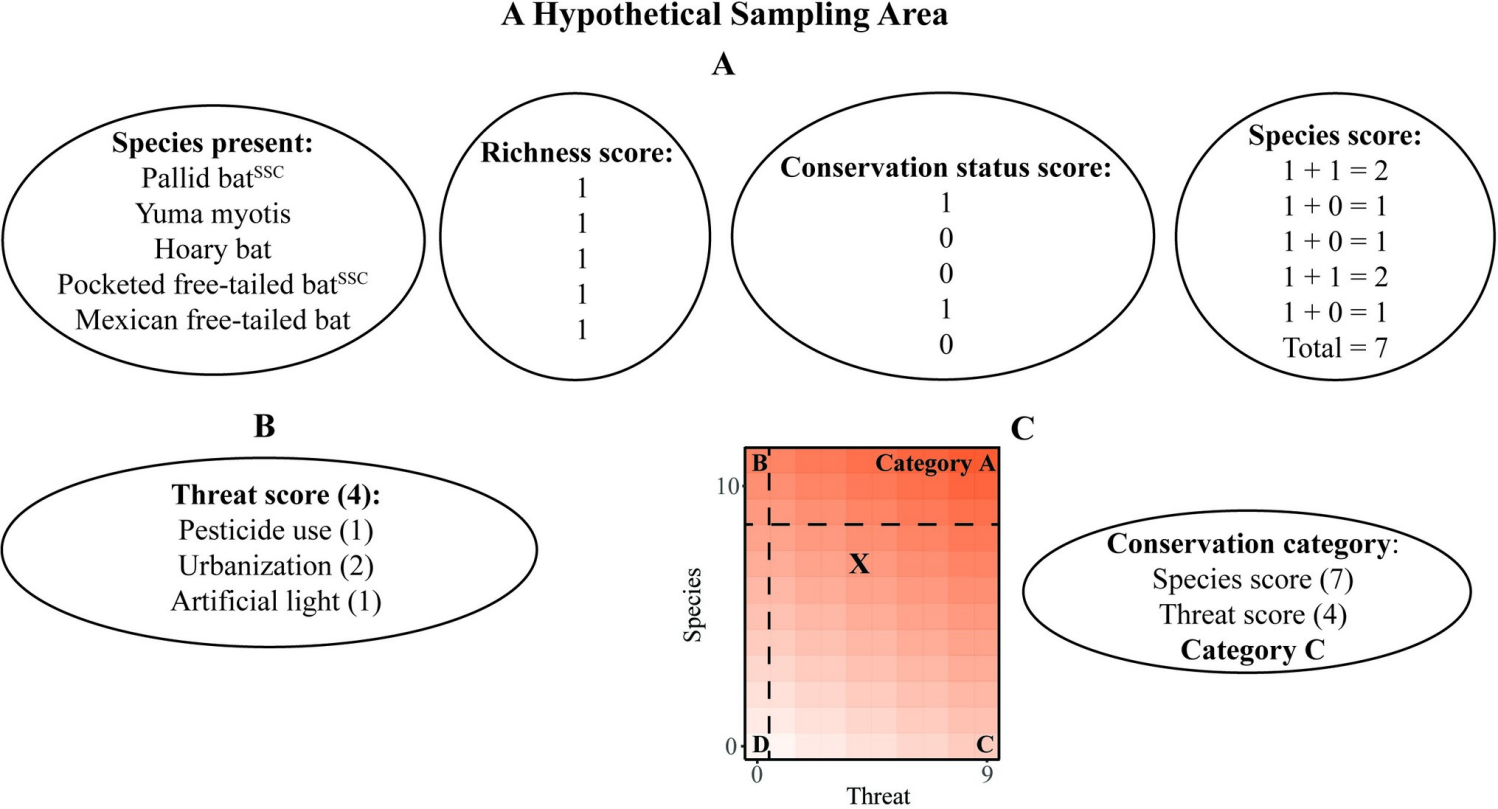
Example of management score computation for a fictional, hypothetical sampling area, with A) species score calculation, B) threat score calculation, C) and the Conservation Category the sampling area was placed in. Numbers in parentheses indicate the score for the adjacent variable.

The species and threat score metrics for each sampling area were visualized on a scatterplot, with all sampling areas present in one of four quadrants to rank sampling areas in terms of species richness, conservation status, and threats for conservation action. The median of all species scores divided the quadrants vertically, while the median of all threat scores divided quadrants horizontally. Scores that were equal to the median were placed in the quadrant with the higher conservation priority. In order from highest conservation priority to lowest, categories (quadrants on the graph) were designated as follows: A) high species score, high foraging threat score; B) high species score, low foraging threat score, C) low species score, high foraging threat score, and D) low species score, low foraging threat score. Sampling areas in Category A rank highest for management action because they have high species richness and several threats exist that could be addressed to help preserve richness. Sampling areas in Category B have high richness and relatively low threat scores, indicating that these sampling areas are relatively intact and may benefit from further monitoring to maintain richness. Category C contains sampling areas with low richness and relatively high threat scores, indicating that management actions could recover species richness by reducing the extent of existing threats. Finally, Category D encompasses sampling areas with both low diversity and low threat scores (Fig 2). Category D sampling areas may lack high species richness due to natural reasons (spatial distribution of habitat, lack of resources, etc.), or threats not identified in the current study. An example of how a Conservation Category is applied for a hypothetical sampling area, including calculation of species and foraging threat scores, is provided in Fig 2.

Sampling areas with high species scores might be related to the presence of lower threat scores. Biologically, this would imply that more species (and higher numbers of individuals) occupy sampling areas that are more intact and have fewer threats. Thus, we used regression analysis to test whether there was a significantly negative relationship between threat scores and species scores.

### Effects of threats on species

Some threats may pose more risk to species than others. To determine whether individual threats were significantly associated with species richness, we fit a GLM with a Poisson family using the function glm() in base R [47, 48]. We included all threats described above, and additionally sampling effort (see below) as predictor variables, with species richness as the response variable. We calculated the Pearson’s correlation coefficient of variables in R to assess multicollinearity in the dataset. Variables with a significant correlation coefficient between them were designated as correlated. In the case of multicollinearity, we built separate models that included one of the correlated variables and the remaining uncorrelated variables in the dataset, until all correlated variables were analyzed. We standardized all predictor variables to have a mean of zero. Standardization allowed us to interpret the relative effect of each predictor variable on species richness using the resultant coefficient for each predictor, while direction (whether the predictor was associated with an increase or decrease in species richness) was interpreted based on whether the coefficient was positive or negative. Significance was evaluated for all statistics using a threshold of P < 0.05.

### Focal species

We sought to identify areas of potential importance to Townsend’s big-eared bat and the pallid bat, two species of conservation concern across their respective breeding ranges, and factors that might be driving their presence and absence. To do so, we ran separate GLMs using the glm() function in base R for each species, as above, with the exception that we fit a GLM with a Binomial family rather than a Poisson family because data were based on the presence or absence of each species in a sampling area [47, 48].

## Results

There was multicollinearity between three threats: artificial lights, urbanization, and unconserved land (unconserved land and urbanization, r = 0.61, P < 0.05, unconserved land and artificial light, r = 0.35, P < 0.05, urbanization and artificial light, r = 0.50, P < 0.05). The GLM that included all variables and did not account for multicollinearity did not identify any threats that were significantly associated with species richness or the presence of Townsend’s big-eared bat or pallid bat (Table 3). Thus, we ran multiple GLMs that each included one of the correlated variables as described in the Methods. We identified significant negative relationships between artificial lights (Z = -3.10, P < 0.05), urbanization (Z = -2.82, P < 0.05), unconserved land (Z = -2.12 P < 0.05), and species richness. Further, urbanization was highest near the coast, and the amount of conserved lands was greatest inland (S2 Fig). Sampling effort was significantly positively associated with species richness (Z = 3.11, P < 0.05). We did not identify a significant negative relationship between any threat variable and the presence of pallid bat. However, artificial lights (Z = -3.29, P < 0.05), urbanization (Z = -2.93, P < 0.05), and unconserved lands (Z = -2.23, P < 0.05) were significantly negatively associated with the presence of Townsend’s big-eared bat. Based on the GLM, survey effort was the largest contributor to the model for species richness (Z = 3.46, P < 0.05) and the detection of Townsend’s big-eared bat (Z = 2.67, P < 0.05) and pallid bat (Z = 1.38, P < 0.05; Table 3).

**Table 3:** Standardized GLM results for total species richness, the pallid bat, and Townsend’s big-eared bat. The absolute value of the coefficient indicates the contribution of each variable to the model. Panel (A) includes all correlated threat variables: unconserved land, urbanization, and artificial light. Panel (B) includes only one of the three correlated threat variables: unconserved land. Panel (C) includes only one of the three correlated threat variables: urbanization. Panel (D) includes only one of the three correlated threat variables: artificial light.

Threat	Pallid				Townsend’s				Richness			
	Est.	SE	Z	P	Est.	SE	Z	P	Est.	SE	Z	P
A												
Number of surveys	5.98E-02	4.35E-02	1.38	0.17	1.49E-01	5.59E-02	2.67	0.01	1.86E-02	5.39E-03	3.46	0.00
Available water	4.376E-07	1.13E-06	0.39	0.70	-4.59E-07	1.30E-06	-0.35	0.72	-1.41E-07	1.68E-07	-0.84	0.40
Unconserved land	1.10E-03	9.02E-03	0.12	0.90	4.93E-03	1.01E-02	0.49	0.62	-2.15E-04	1.25E-03	-0.17	0.86
Urbanization	-4.72E-02	3.26E-02	-1.45	0.15	-6.18E-02	3.91E-02	-1.58	0.11	-3.33E-03	3.06E-03	-1.09	0.28
Artificial light	4.09E-02	4.82E-01	-0.09	0.93	-9.58E-01	5.77E-01	-1.66	0.10	-1.05E-01	6.21E-02	-1.68	0.09
Pesticide use	2.23E+00	1.59E+00	1.40	0.16	1.71E+00	1.76E+00	0.97	0.33	-3.41E-02	2.04E-01	-0.17	0.87
B												
Number of surveys	5.17E-02	3.77E-02	1.37	0.17	9.41E-02	3.90E-02	2.42	0.02	1.61E-02	5.18E-03	3.11	0.00
Available water	9.99E-09	1.09E-06	0.01	0.99	1.08E-06	1.20E-06	0.90	0.37	2.04E-07	1.69E-07	1.21	0.23
Unconserved land	-9.15E-03	7.36E-03	-1.24	0.21	-1.59E-02	7.11E-03	-2.23	0.03	-2.11E-03	9.97E-04	-2.12	0.03
Pesticide use	1.83E+00	1.33E+00	1.37	0.17	1.91E+00	1.31E+00	1.46	0.14	9.44E-02	1.85E-01	0.51	0.61
C												
Number of surveys	6.02E-02	4.32E-02	1.39	0.16	1.42E-01	5.53E-02	2.57	0.01	1.72E-02	5.31E-03	3.24	0.00
Available water	-4.15E-07	1.12E-06	-0.37	0.71	4.52E-07	1.28E-06	0.35	0.72	1.31E-07	1.68E-07	0.78	0.44
Urbanization	-4.39E-02	2.39E-02	-1.83	0.07	-9.06E-02	3.09E-02	-2.93	0.00	-6.33E-03	2.25E-03	-2.82	0.00
Pesticide use	2.18E+00	1.41E+00	1.55	0.12	3.25E+00	1.62E+00	2.01	0.04	9.41E-02	1.83E-01	0.52	0.61
D												
Number of surveys	6.07E-02	3.90E-02	1.56	0.12	1.42E-01	4.74E-02	3.01	0.00	1.90E-02	5.33E-03	3.56	0.00
Available water	-1.02E-08	1.10E-06	-0.09	0.93	7.37E-07	1.23E-06	0.60	0.55	1.69E-07	1.68E-07	1.01	0.31
Artificial light	-4.95E-01	3.79E-01	-1.31	0.19	-1.66E+00	5.04E-01	-3.29	0.00	-1.54E-01	4.97E-02	-3.10	0.00
Pesticide use	1.08E+00	1.30E+00	0.83	0.41	2.40E-01	1.36E+00	0.18	0.86	1.30E-01	1.88E-01	-0.69	0.49

* = significance (P < 0.05).

Out of the 152 original sampling areas, 64 sampling areas included more than one survey and were at least 80% sampled (9.1 +/- 7.6 surveys per sampling area). On average, these 64 sampling areas were 92.4% sampled. We present results only for the 64 sampling areas that were at least 80% sampled.

### Species richness and conservation status

**Fig 3:**
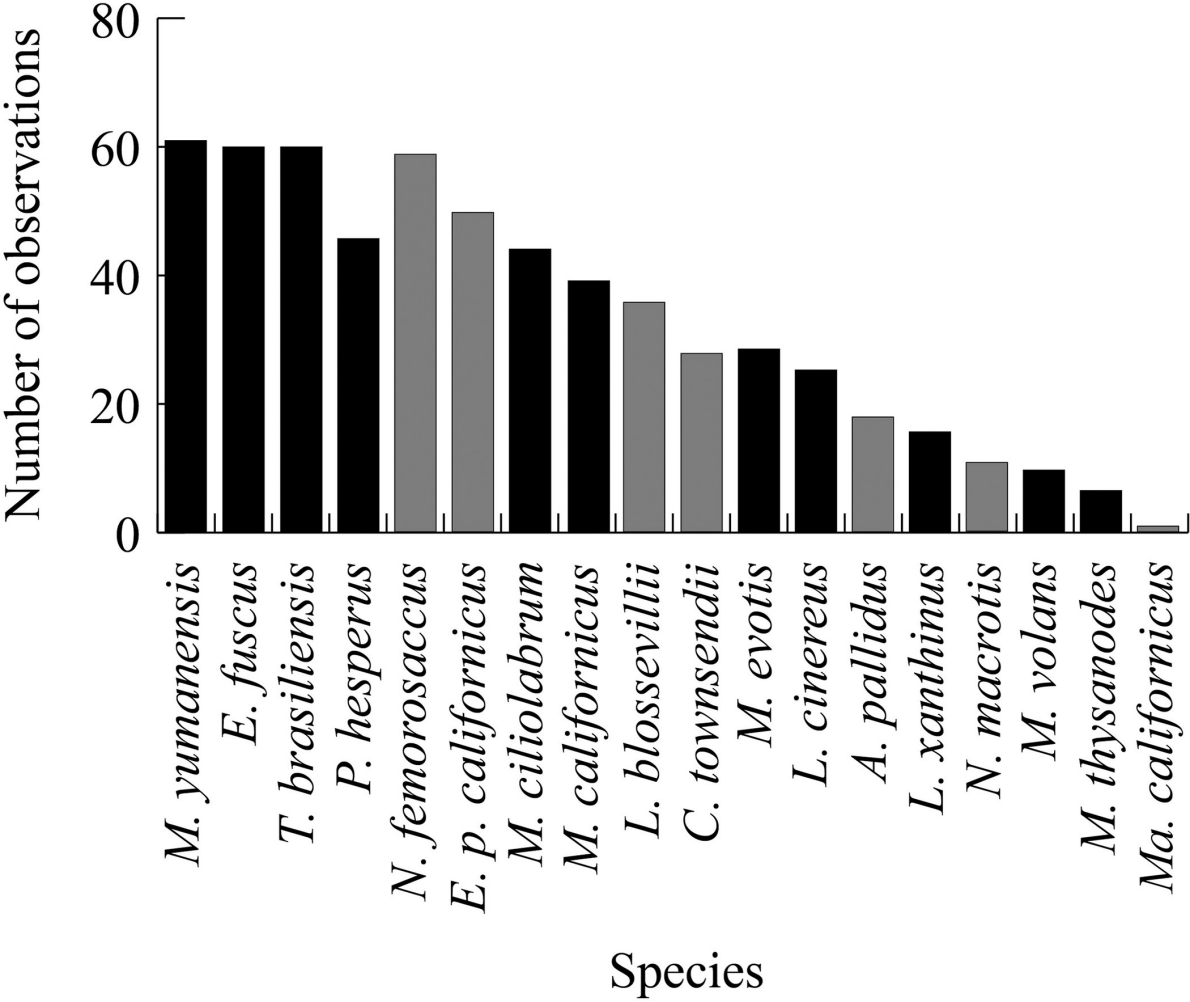
Number of sampling areas, out of 64, at which each bat species was observed. Gray bars indicate a California Species of Special Concern (SSC), while black bars indicate a non-SSC species.

Eighteen species were recorded across 64 sampling areas. Seven of the 18 observed species were designated as SSC, including 18 and 28 sampling areas where pallid bat and Townsend’s big-eared bat were observed, respectively. Yuma myotis (*Myotis yumanensis*, detected at 61 sampling areas), big brown bat (*Eptesicus fuscus*, 60), Mexican free-tailed bat (*Tadarida brasiliensis*, 60), pocketed free-tailed bat (*Nyctinomops femorosaccus*, 59, SSC), and canyon bat (*Parastrellus hesperus*, 53) were observed most often (Fig 3).

Observed species richness ranged from 1 to 15 across all sampling areas (9.3 ± 3.1). Richness was highest (15 species) at Marron Valley, followed by 14 species at each of Hellhole Canyon, Cottonwood Creek Spring, Pamo Valley I, San Felipe Valley I, Sloane Canyon II, and Warner Springs II. The number of species with a conservation status within a given sampling area ranged from 0 to 7 (3.1 ± 1.5). Cottonwood Creek Spring had the highest number of species (7) with a conservation status, followed by El Monte County Park and Marron Valley with 6 species each.

Species scores varied from 0.0 to 21.0 (12.4 ± 4.5, Table 4 and S2 Table). Marron Valley and Cottonwood Creek Spring had the highest overall species score of 21.0 points, a reflection of elevated species richness and number of species with a conservation status in each area. The next-highest species score was found at Warner Springs II, Sloane Canyon II, Hellhole Canyon, and El Monte County Park, each of which scored 19.0 points each.

**Table 4:** Species scores, threat scores, roosting threat scores, and conservation category for each sampling area; sampling areas that contained pallid bat (P) and/or Townsend’s big-eared bat (T) are indicated. The region column indicates where the sampling area is geographically located in San Diego County.

Sampling area	Region	Number	P	T	Species	Threat	Category
Barnett Ranch (West Upland)	North	2		X	15	16	A
Sloane II	South	49		X	19	14	A
Warner Springs II	Northeast	59	X	X	19	12	A
Torrey Pines SNR	West	55			13	16	A
URDS	South	57			16	12	A
Wilderness Gardens	North	64		X	15	13	A
El Monte	Central	15	X	X	19	12	A
Holly South Riparian	Central	21		X	14	14	A
Mission Trails	West	31			11	12	A
North Oaks (Cleveland)	Central	33	X	X	16	13	A
Otay River Valley	Southwest	36	X	X	16	14	A
Parcel North	Northwest	39	X		14	17	A
Parcel South	Northwest	40	X	X	15	16	A
Ramona Grasslands	North	42	X	X	17	14	A
West Sy Creek (West)	Northeast	63	X	X	17	12	A
San Diego River	East	46			13	9	B
Barrett Lake	Southeast	3	X	X	17	8	B
Boden Canyon	North	4			14	9	B
Upper Pine Creek (Cleveland)	East	8			14	8	B
Hellhole	North	19	X	X	19	11	B
Cottonwood (Marron Spring)	South	11	X	X	21	5	B
Dulzura Creek Confluence	South	12		X	13	9	B
West Sy Creek Crossing (East)	Northeast	62		X	17	9	B
Honey Springs Ranch	South	23	X	X	13	7	B
Clover Flat	Southeast	9	X	X	14	10	B
Corte Madera	East	10	X	X	18	7	B
Laguna Troughs	East	26		X	15	7	B
Marron	South	29	X	X	21	6	B
Northeast Dulzura Creek	South	34	X		14	7	B
Pamo II	North	37		X	13	9	B
Pamo I	North	38		X	18	7	B
San Felipe I	Northeast	44		X	18	8	B
Upper San Diego River	Northeast	56		X	16	6	B
Warner Springs I	Northeast	58	X	X	17	7	B
South Parcel (West)	Central	50			8	14	C
West Draw	Northwest	61			5	13	C
Central Draw	North	6			10	16	C
East Oaks (Cleveland)	East	13			12	14	C
Living Coast	Southwest	27			12	17	C
East Sy Creek (East)	Northeast	14			6	12	C
Fairbanks Ranch	West	16			7	17	C
Floodplain	West	18			7	17	C
Fake Pond	North	17			7	16	C
North Creek	North	32			7	16	C
Rancho Canada	North	43			2	11	C
Sweetwater County Park	South	53			7	17	C
West Creek	North	60			7	15	C
SDNHM West Oaks	East	47			10	14	C
Sloane I	Central	48			12	14	C
Oak Oasis	Central	35			9	14	C
Peñasquitos Lower	West	41			12	13	C
South Slope	Northwest	51			9	14	C
Southwest Grove	Northwest	52			11	13	C
Tijuana River Valley	Southwest	54			9	15	C
Hollenbeck Canyon	South	20			7	8	D
Maintenance Shed	South	28			6	10	D
Middle Parcel (South)	Central	30			5	10	D
Cabrillo	Southwest	5			6	8	D
Cibbetts Flat	East	7			11	8	D
San Felipe II	Northeast	45		X	12	7	D
Jamul Creek (Below Kiln)	South	25			9	10	D
Honey Springs Creek	South	22			9	8	D
Jamul Creek (Above Daly)	South	24			11	10	D
Bailey Creek	Northeast	1			9	8	D

### Threat scores

The specific threats within each sampling area, as well as the extent of each threat, varied across the dataset. Threat scores of sampling areas ranged from 5.0 to 17.0 (11.5 ± 3.4, S3 Table). The Living Coast Discovery Center, Fairbanks Ranch, North Parcel, and Sweetwater County Park had the highest threat scores (17.0 points), followed by Barnett Ranch, Fake Pond, North Creek, South Parcel, Torrey Pines, and Central Draw (each scored 16.0 points).

### Conservation categories

**Fig 4:**
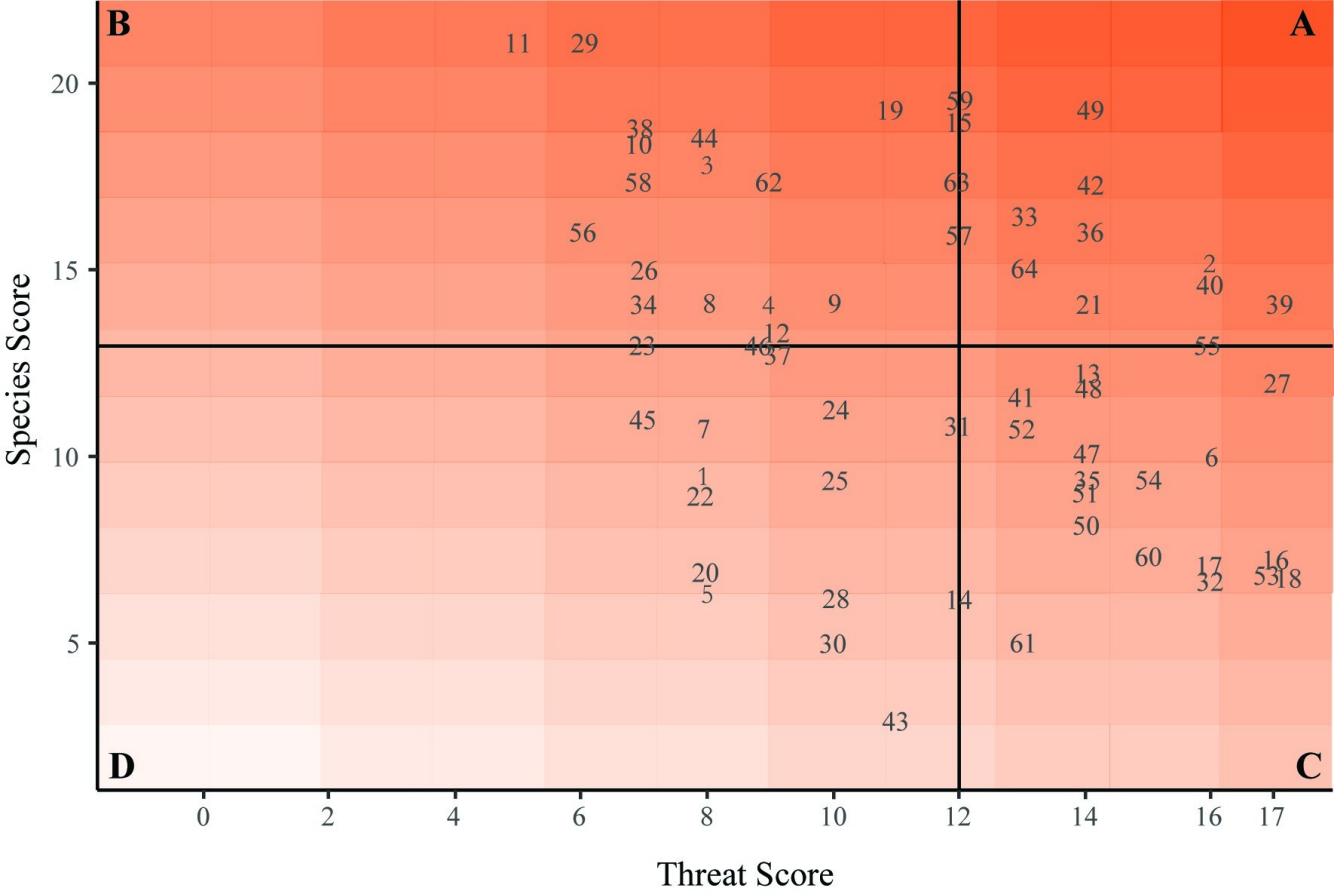
Conservation category of each sampling area based on species scores and threat scores. Category A (high species, high threat); B (high species, low threat); C (low species, high threat); D (low species, low threat). Lines indicate the category thresholds and are based on the median values of the species score (a median of 13.0, horizontal line) and threat score (a median of 12.0, vertical line). Sampling areas plotted directly on a dotted line were assigned to the higher priority category.

Sampling areas were placed into a Conservation Category based on species scores and threat scores (Table 4). Sampling areas within a given category were prioritized based on their locations on the heat map (Fig 4). Based on median thresholds of 13.0 for species score and 12.0 for threat scores, 14 sampling areas were in Category A, 19 in Category B, 20 in Category C, and 11 in Category D. We found a significant negative relationship between threat scores and species scores, indicating that higher threat scores within a sampling area were associated with lower species scores (r = -0.32, *P* < 0.05).

### Townsend’s big-eared bat and pallid bat

We identified significant negative relationships between artificial lights (Z = -3.29, P < 0.05), urbanization (Z = -2.93, P < 0.05), unconserved land (Z = -2.23, P < 0.05), and species presence for Townsend’s big-eared bat, and no significant relationships between any threat and pallid bat. Artificial light (-0.50 and -1.66 for pallid bat and Townsend’s big-eared bat, respectively) and urbanization (-0.04 and -0.09 for pallid bat and Townsend’s big-eared bat, respectively) had the largest coefficients in the negative direction of all threat variables for both species (Table 3). Pesticide use had an insignificant but positive coefficient for both species (2.18 and 3.25 for pallid bat and Townsend’s big-eared bat, respectively; Table 3).

**Fig 5:**
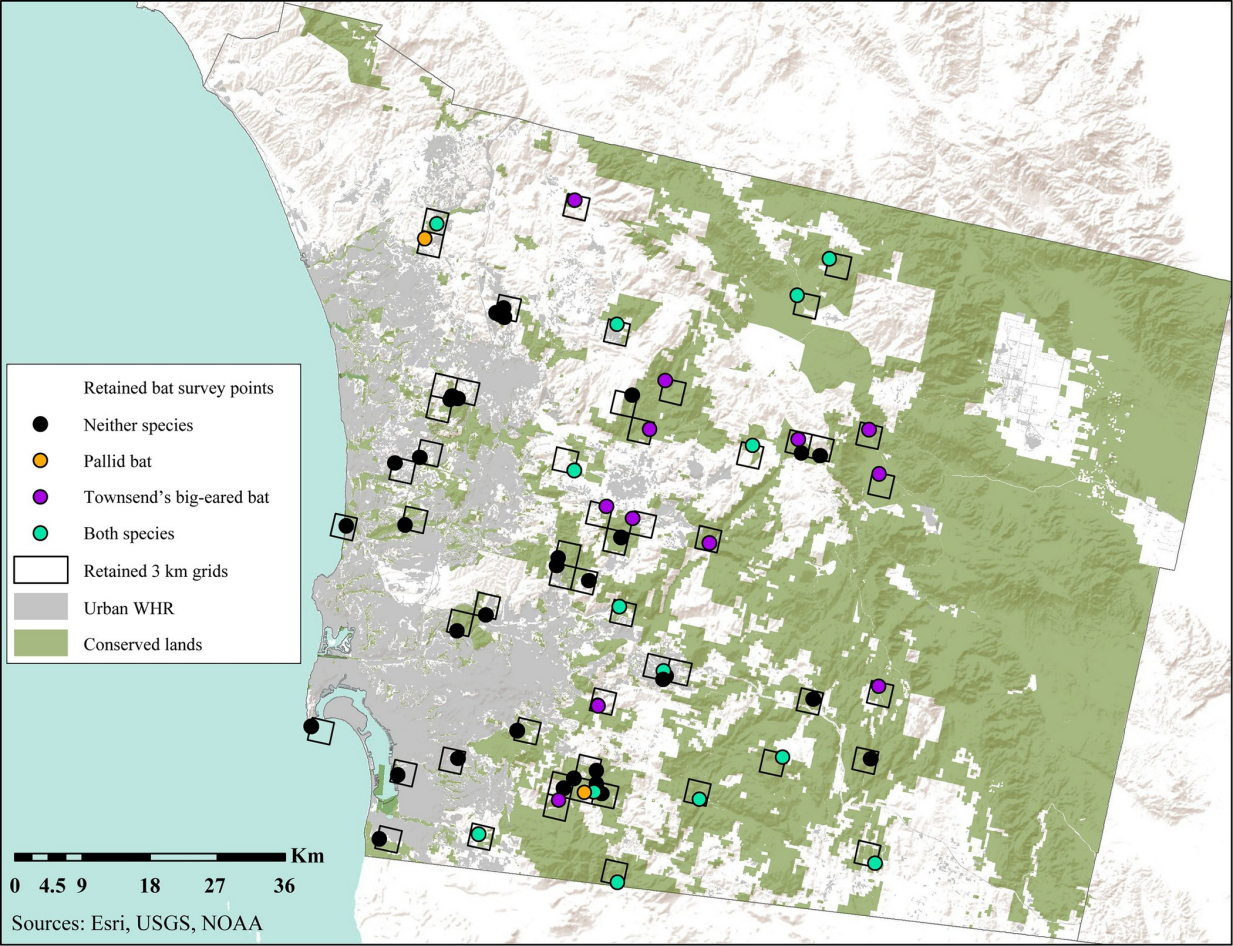
Locations of sampling areas and urbanized areas in San Diego County. Townsend’s big-eared bat and the pallid bat were observed in sampling areas filled in purple and orange respectively, while sampling areas where both species were observed are filled in turquoise. Both species were largely absent from sampling areas with high proportions of urbanization in the western part of the county [55–59].

Townsend’s big-eared bat and/or pallid bat were observed within 27 and 17 sampling areas respectively (Table 4). Seven sampling areas with at least one survey in which both pallid bat and Townsend’s big-eared bat were observed were in Conservation Category A, the highest Conservation Category, while eight were in Conservation Category B (Table 4). Pallid bat and Townsend’s big-eared bat were not observed along the coast, where urbanization was highest (Fig 5).

## Discussion

Our study, focused on the bat community in San Diego County, provides a spatial approach that can leverage diverse datasets to prioritize areas for wildlife conservation and management. Of the 41 species of bats that occur in the United States, the present study compiles documentation of 18 in San Diego County, 8 of which are SSC (Table 1). An essential part of a conservation plan for so many bat species should focus on the protection of areas of the landscape used by bats, not only physical locations where individuals are identified [68, 69]. The current study accounted for threats, species richness, and conservation status to conserve bat species in San Diego County and provides an objective framework for future bat conservation studies at the community level. Further, our study provides an example of how a spatial approach can harmonize information gleaned from multiple sources of data, and consistently with other approaches to wildlife conservation, implements a ranking system to enable conservation action at the regional level [70, 71].

### The threat landscape

Threats, if realized, can have drastic effects on the bat community. We outline potential management options that could be implemented to mitigate each threat that contributed to threat scores, which include the protection of bat roosts and the protection and provision of critical resources, in Table 5. Additionally, more long-term roosting surveys could assist in documenting and understanding bat occurrences in addition to species richness surveys. For example, many sampling areas likely contain roosting sites, which are critically important to bat species, and a single exclusion event in which a landowner might seek to remove bats from unconserved land might displace thousands of individuals [23]. As urbanization and development in general are increasing rapidly around the world, and as a result, bats become more reliant on human-built structures such as bridges and buildings, effective education of the public is a critical management strategy [19]. In fact, roost disturbance and destruction of caves and human-made structures are key drivers of the declines of bat populations across the world [72]. Standardized, localized datasets that include estimates of roosting species richness and abundance would provide management with a critical tool to assist prioritization of management areas. For example, two sampled areas with equivalent species richness and comparable threat scores may appear to require equal prioritization for management, but if one sampling area contains a high abundance of roosting species, and the other does not, the sampling area with the high abundance of roosting species could be prioritized.

**Table 5:** Possible management options for landscape-level threats.

Threat descriptor	Potential management options
Unconserved land	Sampling area is on unconserved land. Formation of an agreement with the current landowner and/or humane removal of bats, although a last resort, might be necessary if bats are under threat of intentional exclusion or extermination.
Pesticide use	In-depth study of the effects of insect control (and the chemicals used) on bats, and explicit testing of the hypothesis that pesticide use lowers food availability and quality. Depending on results, reduction or cessation of pesticide use could be implemented on conserved lands.
Lack of open water	Restoration and/or maintenance of seasonal water sources (i.e., water troughs, tanks, ponds, and pools) during the dry season, construction of permanent water sources where they are lacking.
Artificial lights	Partial night-lighting, where artificial lights are powered off during certain hours of the night, might be effective for some species [30]. Removal of artificial lights from areas inhabited by bats. In-depth study of the effects of artificial lighting on bats, and identification of species that benefit and are negatively impacted by artificial lighting.
Urbanization	Consider conserving lands where bats are present, especially when encroaching into lesser-developed foothill areas. If urbanization must proceed, or it has already occurred, mitigation measures likely to assist bat populations can include a reduction of artificial lighting, restoration of riparian habitat, and removal of invasive vegetation.

GLMs identified significant negative associations between artificial lights (Z = -3.10, P < 0.05), urbanization (Z = -2.82, P < 0.05), unconserved land (Z = -2.12 P < 0.05), and species richness, and these results are consistent with the findings of prior work. Urbanization likely makes foraging more difficult for bats that roost in human-made structures [73], and bat activity declines with increases in urban density [63]. Finally, artificial lights present a foraging advantage for some species, but not for most, as light pollution is generally considered a threat to bat biodiversity [74].

No threat was significantly associated with the presence of pallid bat, although artificial lights (Z = -3.29, P < 0.05), urbanization (Z = -2.93, P < 0.05), and unconserved land (Z = -2.23, P < 0.05) were significantly negatively associated with the presence of Townsend’s big-eared bat (Table 3). It is possible that additional threat variables that are significantly negatively associated with species richness or the presence of pallid bat and/or Townsend’s big-eared bat in San Diego County would be revealed by the GLM with additional sampling.

The threats identified in the current study, although critically important to address in San Diego County and elsewhere, are a subset of the numerous threats faced by the bat community across the world. Climate change may be a significant threat to some bat species. Shifts in the climate may disrupt migratory behavior [75], foraging behavior [76], survival and reproduction [77], and disrupt food availability for pollinating species via changes in flowering phenology [78]. Other major threats relate to habitat loss. For example, logging and agriculture are ranked as the two most common threats faced by the worldwide bat community [19]. Global and regional collaboration among land managers, scientists, and landowners are needed to address these threats and ensure the long-term sustainability of the worldwide bat community. Indeed, the significantly negative relationship observed between species richness and sampling areas on unconserved land highlight the need for additional dialogue between public land managers and private landowners. As threats are exacerbated over time, especially with increased development and human activity across San Diego County [22], sampling areas, especially those with high threat scores, may lose species richness, emphasizing the importance of continued monitoring.

### Limitations and future sampling

Although the present study identified and proposed specific management action for several sampling areas across San Diego County, several issues still need to be addressed. Bat survey methods and technologies have improved over time and uniformity of data and survey effort can be difficult to achieve with long-term datasets. Given the cryptic nature of bats, under sampling is a common issue, and previous work recommends simultaneous implementation of numerous sampling techniques to maximize sampling efficiency, as some methods are more effective than others at species-specific detection [72, 79]. Most recommended techniques were implemented in our study, although not always during the same sampling event, including use of bat detectors, mist nests, and roost surveys.

In the current study, existing survey data and survey efforts were not evenly or randomly distributed. We identified survey gaps across San Diego County and highlighted where additional data are needed, particularly in the northern and eastern portions of San Diego County (Fig 1). Further, several of the sampling areas in the current study were under sampled and discarded from analysis to avoid analysis of areas that were sampled unevenly. In addition to filling in gaps in sampling effort, future survey efforts could incorporate habitat suitability models for species of interest to identify under-surveyed areas with high predicted presence and test associations between habitat, climatic, and landscape features with predicted species presence. Despite the limitations of our study, the standardization of multiple disparate sources across a larger area can still allow conservation planners to apply all available data gathered over extended time periods to conservation management and decision making [71, 80, 81].

### Townsend’s big-eared bat and pallid bat

Both the Townsend’s big-eared bat and pallid bat were historically common throughout their respective ranges, but that is no longer the case [32, 33]. Disturbance of roosting sites is a significant threat to each species, and climate change may be exacerbating the challenges these species face. For example, a continued contraction of suitable habitat is expected over time due to climate change [82]. Because both species are roost-limited, and individuals are often observed foraging, and not at their roosts, we suggest management action for these species be based on prioritization of sampling areas where each species was observed and where threats were scored highest. With the incorporation of the 3 km buffer used in the current study, roosts are likely to be within the buffered area due to the relatively short homing ranges of each species in the area [29, 51–54]. Further, for pallid bat, a foraging specialist, available foraging habitat is equally important to roosting habitat and appears to be a limiting resource for the species in the region. The pallid bat tends to forage in low gradient habitats where human land-use practices are the most prevalent [22, 32, 35]. Pallid bat may also be rarer in the region than Townsend’s big-eared bat (observed in 27% and 42% of sampling areas, respectively), and potentially more vulnerable. Other recent survey efforts also highlighted the recent rarity of pallid bat: this species is now rare in several areas where it was previously common [83].

It is surprising that neither Townsend’s big-eared bat nor pallid bat was observed along the coast or within inland areas where urbanization was highest (Fig 5, [71]), and while the presence of Townsend’s big-eared bat was negatively associated with urbanization, the presence of pallid bat was not, although this result may change with increased sampling. Urbanization in general is harmful to bat populations, and although extensive mitigation of already-urbanized areas might not be realistic (except for minor alterations and the addition of bat-friendly habitat features), as development of urban areas in the foothills continues, mitigation strategies for bat conservation, especially for Townsend’s big-eared bat and likely pallid bat, should be kept in mind. Formation of bat-friendly habitat that is at least equal in size to any new urban area, for example, artificial bodies of water and patches of woodland habitat conserved and restored within the urban-wildlife matrix, might provide an effective mitigation measure [84]. Expanding existing blocks of urban areas rather than creating new urban patches in undeveloped habitat could be better for maintaining populations of Townsend’s big-eared bat, pallid bat, and biodiversity in general [71, 84, 85]. Recently, Townsend’s big-eared bat and the pallid bat were observed at military bases along the coast in San Diego County, possibly highlighting the importance of military installations and the preservation of land that species occupy (D. Stokes pers. observation.).

Pallid bat and Townsend’s big-eared bat exhibit differences in roosting preferences. For example, Townsend’s big-eared bat almost exclusively roosts in caves or abandoned mines (A. Calvert, National Park Service, pers. comm., 12 January 2024), while pallid bat exhibits a wide range of roosting preferences, including in buildings and bat houses [86]. The latter may explain why the presence of pallid bat was not significantly negatively associated with urbanization, unlike Townsend’s big-eared bat. The ability of the pallid bat to exploit artificial roosting structures has notable conservation implications: while the main priority should be to preserve the natural habitat of the pallid bat and Townsend’s big-eared bat, a subset of bat species would benefit from the installation of bat houses in situations where urbanization is irreversible [63].

### Prioritization of conservation needs

Complex conservation issues require a more regional approach, where the most pressing conservation needs might vary across geographic space. The SDMMP seeks to identify county-specific conservation issues for taxa on conserved lands, including bats [7]. Consistent with the goals of the SDMMP, the framework presented here took advantage of empirical data to identify areas of high conservation value where actions could improve bat habitat across San Diego County. The conservation prioritization categories formed for each sampling area were comprised of delineated species and threat scores that were computed for each sampling area, which enables a management entity to choose how to take specific action based on feasibility, as determined at the local level.

The framework introduced here presents a dynamic strategy to implement for bat conservation management, and can be used within any environment or ecosystem to highlight areas of conservation concern. Bat habitat extends far beyond a single location, and the approach in the current study enables the inclusion of multiple portions of the landscape, which is critical for a bat management plan to be effective. Due to its flexible and detail-oriented nature, our approach is applicable to bat conservation plans in San Diego County and wherever bats occur. Our approach can ensure that long-term datasets are evenly sampled prior to quantification of threats to bats and promote subsequent management action that reflects bat community composition across sampling areas. Finally, the strategies presented here need not be limited to bats and are applicable to any species that require management attention at the local level.

## Supporting information

S1 Fig Locations of sampling areas that were analyzed after removing under-sampled areas, and average light pollution score within a given sampling area, based on Bortle Class [55–59]. (TIF)

S2 Fig Species richness of each retained sampling area, across urbanized and unurbanized areas, and across conserved versus unconserved lands [55, 56]. (TIF)

S1 Table Sampling, locality, and species score information. Survey methods were as follows: A = anabat, E = the unaided ear, V = visual, M = mist net.
(XLSX)

S2 Table Species accumulation curve and species richness estimation of each sampling area. (XLSX)

S3 Table Locality data for Generalized Linear Models (GLMs). (XLSX)
