# Supplementary figures and images for: Quantification of threats to bats at localized spatial scales for conservation and management
Source: PLoS One. 2024 Oct 9;19(10):e0310812. doi: 10.1371/journal.pone.0310812 (PMC11463755; doi:10.1371/journal.pone.0310812)

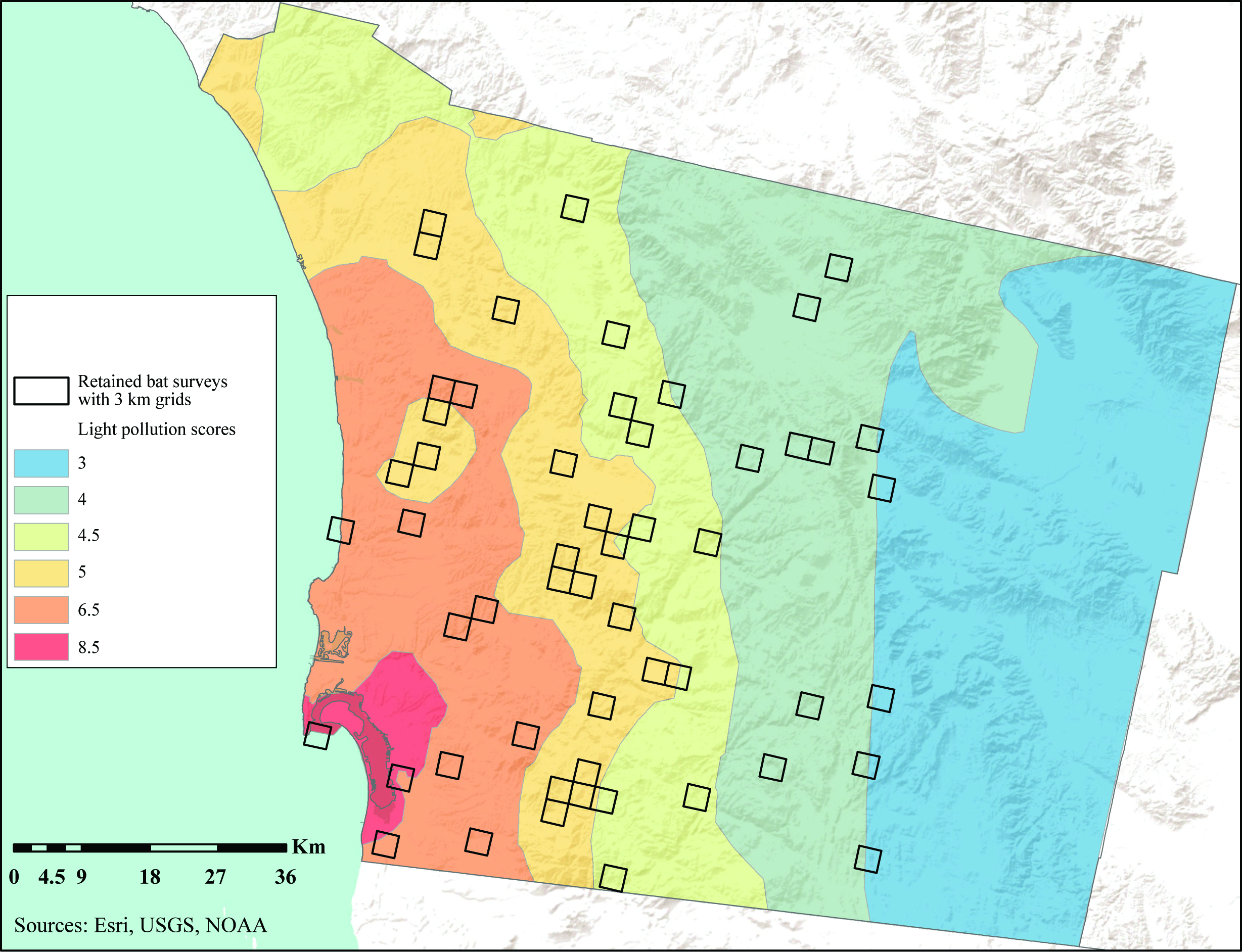

Supplement: S1 Fig — (TIF) [file pone.0310812.s001.tif]

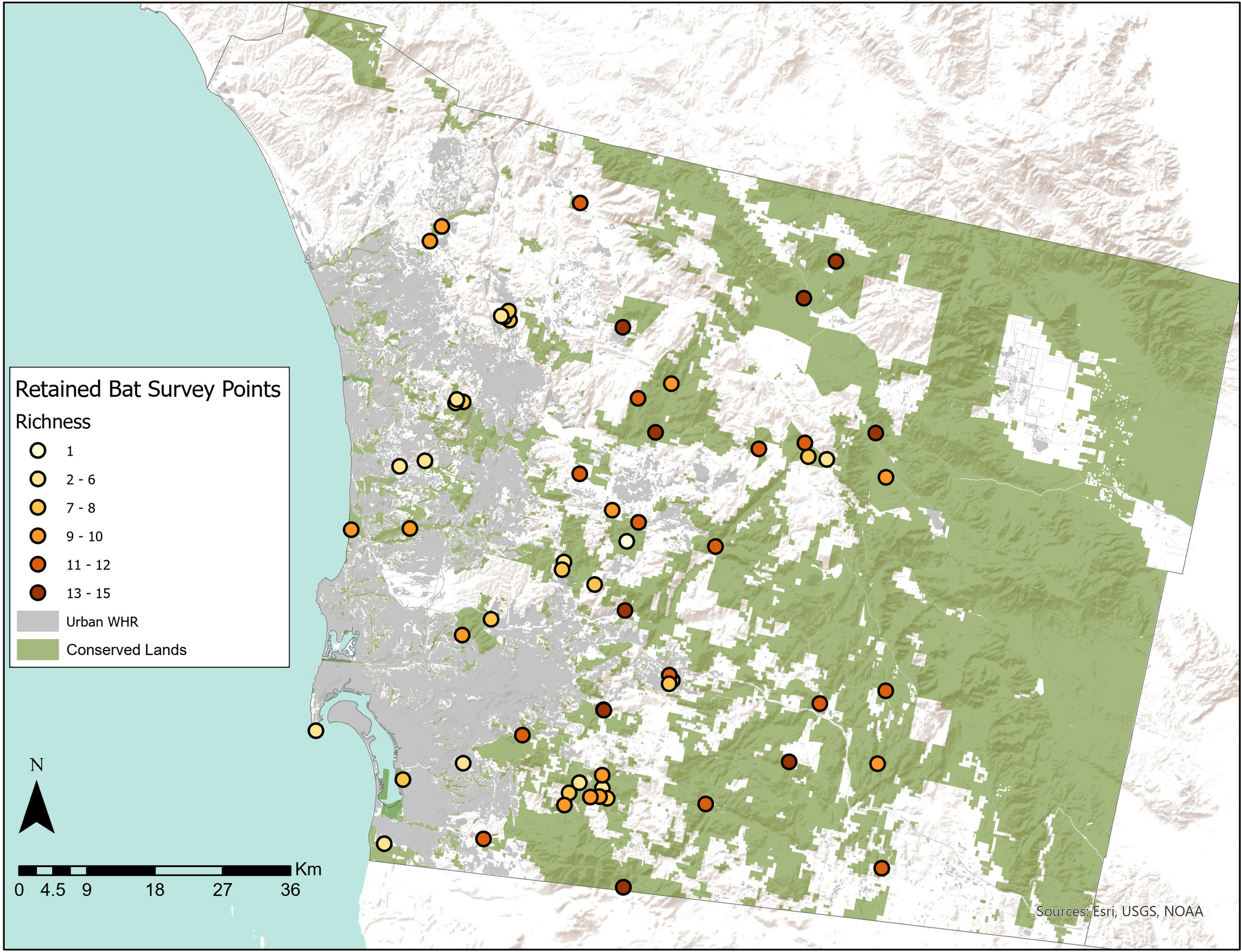

Supplement: S2 Fig — (TIF) [file pone.0310812.s002.tif]
